# The Youngest Doctor

**Published:** 1891-02

**Authors:** 


THE YOUNGEST DOCTOR.—The Southern Medical Society which met recently at Atlanta, Georgia, elected to honorary membership Master Venner Tensch, of Fort McPherson, Ga. Although only five years old and a kindergarten student, he has a phenomenal knowledge of anatomy, lectures upon which subject he attends, and charts relating to which he studies instead of pictures. In an exhibition before the society he gave the names of all the bones in the body, and also described their functions, divisions, tuberosities, tubercles, etc. He appeared fully to appreciate the honor conferred upon him, and thanked the president and gentlemen for their action. The child is known as Doctor Albert, and chooses his friends from among the doctors and medical students instead of playing tops and marbles with the boys.

